# Knowledge and awareness of nonpharmacist salespersons regarding over-the-counter drug use in patients with chronic kidney disease in Japan

**DOI:** 10.1371/journal.pone.0213763

**Published:** 2019-03-20

**Authors:** Yuki Kondo, Yoichi Ishitsuka, Nobuhiro Kawabata, Nobuhide Iwamoto, Risa Takahashi, Yuki Narita, Daisuke Kadowaki, Sumio Hirata, Satoru Uchino, Tetsumi Irie

**Affiliations:** 1 Department of Clinical Chemistry and Informatics, Graduate School of Pharmaceutical Sciences, Kumamoto University, 5–1 Oe-honmachi, Chuo-ku, Kumamoto, Japan; 2 Kagoshima Pharmaceutical Association, 2-8-15 Yojiro, Kagoshima, Japan; 3 Department of Clinical Pharmacology, Faculty of Pharmaceutical Sciences, Kumamoto University, 5–1 Oe-honmachi, Chuo-ku, Kumamoto, Japan; 4 Laboratory of Clinical Pharmaceutics, Faculty of Pharmaceutical Sciences, Sojo University, 4-22-1 Ikeda, Kumamoto, Japan; 5 Center for Clinical Pharmaceutical Sciences, Faculty of Pharmaceutical Sciences, Kumamoto University, 5–1 Oe-honmachi, Chuo-ku, Kumamoto, Japan; Postgraduate Medical Institute, INDIA

## Abstract

**Introduction:**

Patients with chronic kidney disease (CKD) depend on advice from healthcare professionals to avoid using unsuitable over-the-counter (OTC) drugs. Recently, qualified, registered nonpharmacist salespersons became licensed to sell OTC drugs in Japan. However, registered salespersons’ knowledge and awareness of precautions regarding unsuitable OTC drugs for use in patients with CKD are unclear.

**Objectives:**

This study aimed to clarify the awareness, knowledge, and implementation of precautions by registered salespersons regarding OTC drugs used by patients with CKD. Additionally, we evaluated the change of registered salespersons’ knowledge and awareness of this topic generated by a pharmacist intervention.

**Methods:**

A questionnaire survey and pharmacist intervention were applied to 175 registered salespersons. The intervention comprised a 50-minute lecture imparted by a pharmacist who was trained in nephrology. The knowledge, awareness, and implementation of precautions by participants with respect to nonsteroidal anti-inflammatory drugs (NSAIDs) and antacids were evaluated before and after the intervention.

**Results:**

Approximately half of the registered salespersons reported previous experience with selling OTC drugs that were inappropriate for patients with CKD (NSAIDs, 48.0%; antacids, 39.7%). Few participants recognized the need to check renal function when selling those drugs to such patients (NSAIDs, 25.7%; antacids, 47.5%). The registered salespersons’ awareness and knowledge were significantly higher after the intervention than before it.

**Conclusion:**

The results indicate that before the intervention, the registered salespersons had low levels of awareness and knowledge regarding OTC drug use in patients with CKD despite having prior experience selling unsuitable OTC drugs. However, the pharmacist intervention improved the registered salespersons’ awareness and knowledge. The educational program for registered salespersons might be necessary to prevent inappropriate OTC drug use by patients with CKD.

## 1 Introduction

Chronic kidney disease (CKD) is an important and common health problem, and its incidence is increasing worldwide. The estimated overall prevalence of CKD (glomerular filtration rate < 60 mL/min/1.73 m^2^) in adults (aged ≥18 years) is increasing exponentially in Japan, particularly among the aging population [1]. Patients with CKD have a higher incidence of adverse drug events than patients without the disease [2–4]. Thus, it is necessary to pay close attention to medication use when treating patients with CKD, including the use of over-the-counter (OTC) drugs.

Although pharmacists can contribute to a reduction in the incidence of adverse drug events in patients [5], in 2009, it became possible for qualified nonpharmacists (“registered salespersons”) to sell OTC drugs in Japan [6]. OTC drugs are classified into three categories in Japan according to risk level. Pharmacists can sell all classes of OTC drugs. Although registered salespersons are qualified to sell Class 2 (relatively high risk) and Class 3 (relatively low risk) drugs, these classes of OTC drugs include nonsteroidal anti-inflammatory drugs (NSAIDs) and antacids. Unsupervised use of NSAIDs [7] and antacids [8] should be avoided by patients with CKD, given their potential adverse effects in such patients. The package inserts of OTC NSAIDs (such as ibuprofen) contain a warning against their use in patients with renal disease. Thus, it is important that registered salespersons and pharmacists check customers’ renal function status before selling NSAIDs and antacids to them. Additionally, important notices about pharmacotherapy in patients with renal insufficiency are described in the creation guide for the qualifying examination for registered salespersons. Thus, registered salespersons should understand the appropriate use of OTC drugs in patients with renal disease. However, little is known about registered salespersons’ knowledge, awareness, and implementation of precautions regarding OTC drugs used in patients.

In this study, we conducted a questionnaire survey to assess the knowledge, awareness, and implementation of precautions by registered salespersons regarding OTC drug use in this population. Additionally, we evaluated the effectiveness of a pharmacist intervention at improving registered salespersons’ knowledge and awareness regarding the use of OTC drugs by this susceptible population.

## 2 Materials and methods

### 2.1 Study design

The questionnaire survey and pharmacist intervention were conducted via continuation training for registered salespersons by the Kagoshima Pharmaceutical Association. Invitations to the continuation training program were sent to all members of the Kagoshima Pharmaceutical Association, and participation was voluntary. However, registered salespersons are required to participate in 12 hours of lecture programs per year, and there are few opportunities for registered salespersons to attend the programs. Therefore, this program was almost compulsory for many registered salespersons who worked with members of the Association. The presurvey consisted of questions about the participants’ baseline characteristics, knowledge, and awareness. The items in the questionnaire are listed in Table 1. All participants completed the presurvey before participating in the intervention. The participants were not notified of the contens of the lecture beforehand. After the intervention, the participants completed the same questionnaire survey. The Ethics Committee of Kumamoto University approved this study (no. 886). Verbal informed consent was obtained from all participants. The data were analyzed anonymously.

**Table 1:** Questionnaire for registered salespersons.

Information Requested	Information Obtained
Characteristics of participating registered salespersons	Sex, age, work experience, type of workplace, handles merchandize/drugs at their workplace
Experience with selling unsuitable drugs for patients with CKD	Yes/No questions, (selling NSAIDs, selling antacids)
Awareness of pharmacotherapy for patients with CKD	Three items, using a 5-point Likert-type scale
Confirmation items are required to sell NSAIDs	Choose three from the list: upset stomach, aspirin-induced asthma, long-term use, kidney disease, liver disease, drinking, smoking, concomitant medication
Confirmation items are required to sell antacids	Choose three from the list: hypertension, asthma, long-term use, kidney disease, liver disease, drinking, smoking, concomitant medication
Examination questions about risk factors for CKD	Choose three from the list: depression, liver disease, diabetes, hypertension, Crohn's disease, aging
Examination questions about pharmacotherapy of patients with CKD	True/False questions, three items
Examination questions about unsuitable drugs for patients with CKD	Choose four from eight drugs in the list
Participants’ satisfaction with the pharmacist intervention	Five-point Likert-type scale

CKD, chronic kidney disease; NSAID, nonsteroidal anti-inflammatory drug.

### 2.2 Questionnaire

The questionnaire used in the study was created by licensed pharmacists (YK, YI, and NK). To assess the validity and reliability of the questionnaire, we performed a pilot survey among a small sample of pharmacy students. The questionnaire was revised according to the results of the pilot survey. Because the questionnaire was written in Japanese, the translated version is shown in S1 File.

### 2.3 Pharmacist intervention

The pharmacist intervention comprised a 50-minute lecture, the objective of which was to impart sufficient skills for the participants to manage OTC drug use by patients with CKD. The lecture consisted of three parts: the epidemiology of CKD in Japan, the OTC drugs that should be avoided in patients with CKD, and the triage of patients with CKD. The speaker was a pharmacist trained in nephrology (YK), and he used the Prezi presentation tool (Prezi Inc., San Francisco, CA, USA) [9,10]. The details of the lecture contents are shown in S1 Table.

### 2.4 Statistical analysis

All statistical analyses were performed with JMP Pro 13 (SAS Institute Inc., Cary, NC, USA). Comparisons of categorical variables between before and after the intervention were performed using McNemar’s test. Comparisons between continuous variables between before and after the intervention were performed using the Wilcoxon signed-rank test. Significance was set at *p* < 0.05 for all analyses.

## 3 Results

### 3.1 Participants’ characteristics

The characteristics of the participating registered salespersons are shown in Table 2. A total of 179 registered salespersons completely responded to both surveys (response rate: 91.8%). Of those, 161 (89.9%) were women. Their mean age and working experience were 41.6 years and 8.8 years, respectively. Of all respondents, 172 (96.1%) registered salespersons worked in pharmacies that dispensed prescription medications. Of the participating registered salespersons, 169 (94.4%) dealt with OTC drugs, 155 (86.6%) sold quasi-drugs (a unique product classification in Japan, including energy drinks containing taurine and/or some vitamins, skin whitening products, and fluoridated toothpaste), 87 (46.6%) dealt with cosmetics, and 123 (68.7%) sold functional foods and dietary supplements.

**Table 2:** Characteristics of participating registered salespersons.

Characteristic	
Response rate, n (%)	179/195 (91.8)
Sex, n (%)	
Male	18 (10.1)
Female	161 (89.9)
Age in years, mean ± SD	41.6 ± 10.2
Work experience in years, mean ± SD	8.8 ± 6.9
Type of workplace, n (%)	
Pharmacy (deals with prescription medicine)	172 (96.1)
Drug store (does not deal with prescription medicine)	7 (3.9)
Deal with goods in their workplace, n (%)	
OTC drugs	169 (94.4)
Quasi-drugs (e.g., cough drops)	155 (86.6)
Cosmetics	87 (46.6)
Functional foods and dietary supplements	123 (68.7)

SD, standard deviation; OTC, over-the-counter

### 3.2 Participants’ experience and knowledge

Approximately half of the registered salespersons (n = 86, 48.0%) sold NSAIDs, and 71 (39.7%) sold antacids to patients with CKD (Table 3).

**Table 3:** Registered salespersons’ experience with selling unsuitable drugs for patients with CKD.

Medicine	
NSAIDs, n (%)	
Yes	86 (48.0)
No	93 (52.0)
Antacids	
Yes	71 (39.7)
No	108 (60.3)

CKD, chronic kidney disease; NSAID, nonsteroidal anti-inflammatory drug

Only 46 (25.7%) and 85 (47.5%) of the registered salespersons recognized the need to check patients’ renal function status when selling NSAIDs and antacids, respectively. In contrast, 175 (97.8%) registered salespersons recognized the need to check patients’ renal function when selling both NSAIDs and antacids after the intervention (Table 4).

**Table 4:** Registered salespersons’ recognition of the need to check renal function when selling NSAIDs and antacids to patients with CKD.

Medicine	Pre-intervention n (%)	Post-intervention n (%)	*p* value
NSAIDs Chose to check renal function	46 (25.7)	175 (97.8)	< 0.001 ^a^
Antacids Chose to check renal function	85 (47.5)	175 (97.8)	< 0.001 ^a^

CKD, chronic kidney disease; NSAID, nonsteroidal anti-inflammatory drug.

^a^ McNemar's test.

### 3.3 Effects of pharmacist intervention

**Fig 1:**
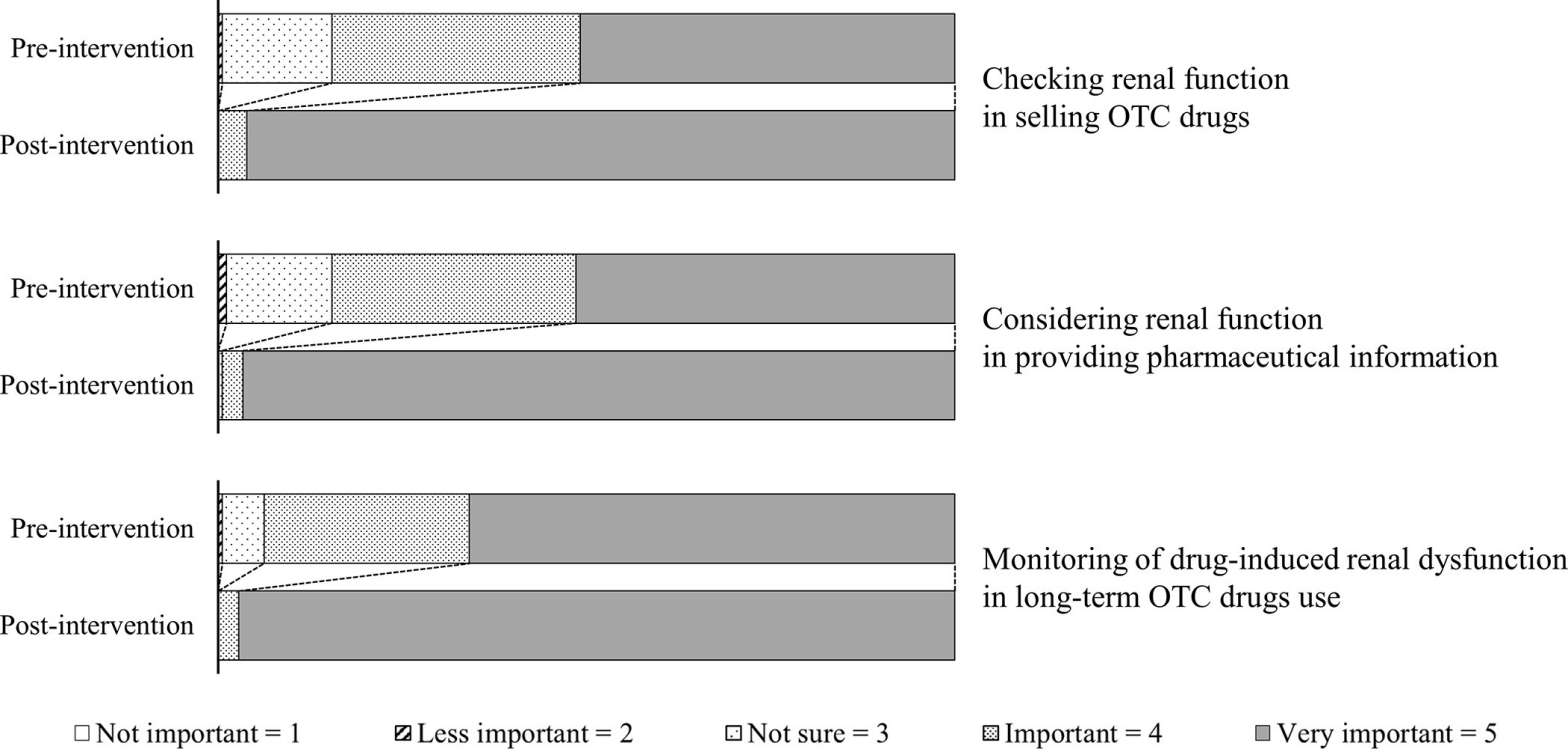
Registered salespersons’ awareness of pharmacotherapy for patients with chronic kidney disease. OTC, over-the-counter.

**Fig 2:**
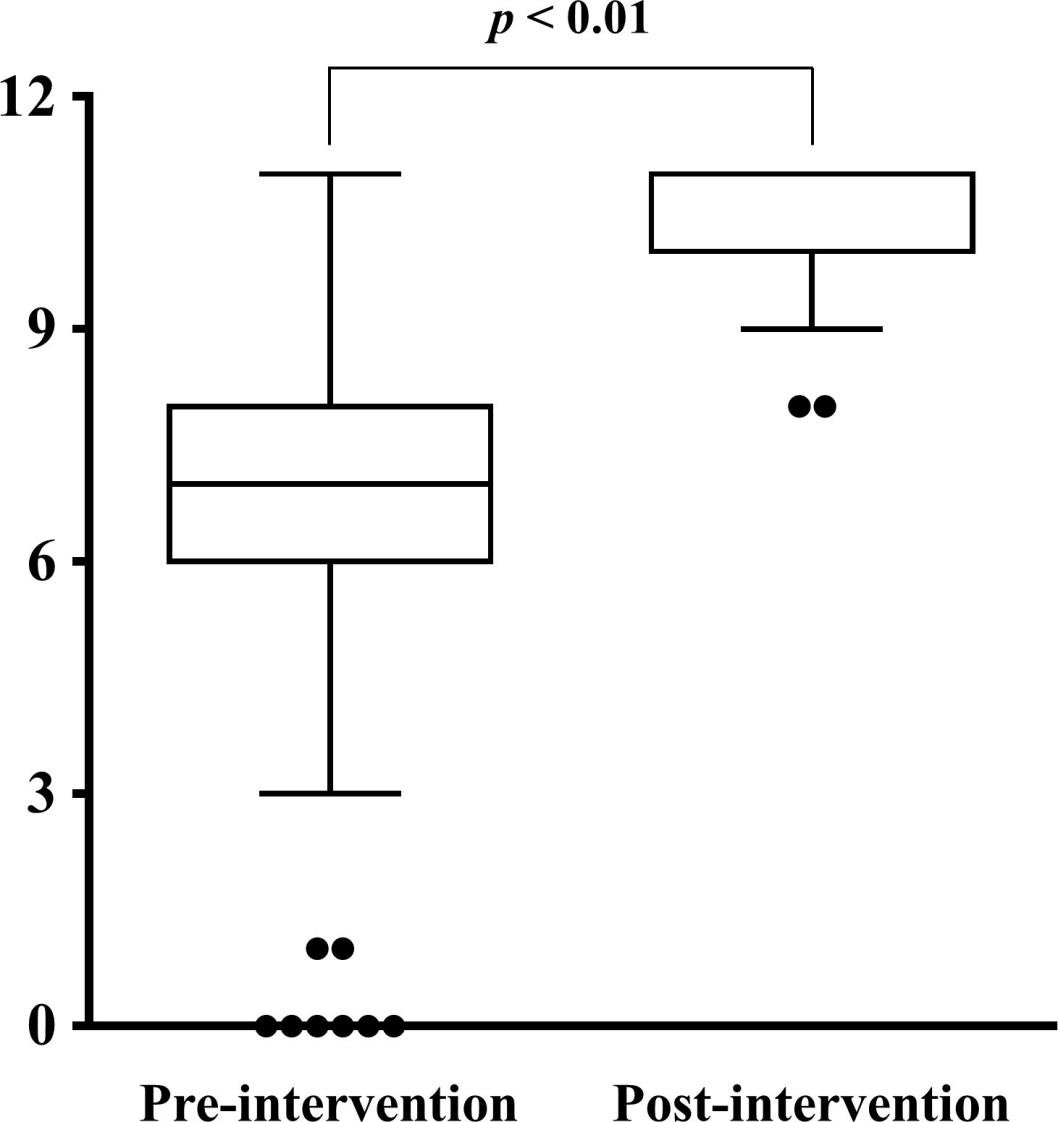
Registered salespersons’ knowledge about OTC drugs in patients with CKD. The box plot shows registered salespersons’ knowledge scores (maximum score: 11, n = 197). In the box plots, the median is denoted by the center horizontal line. The bottom and top of the box show the 25% and 75% rankings, respectively, denoting the interquartile range. The minimum and maximum rankings are denoted by the lower and upper whiskers, respectively. Outliers are denoted by circles. CKD, chronic kidney disease; OTC, over-the-counter.

Post-intervention awareness was significantly higher than that measured in the pre-intervention assessment. The median Likert scale score distributions (with interquartile range) are shown in Fig 1. The results of the post-intervention knowledge assessment are shown in Table 5 and Fig 2.

**Table 5:** Registered salespersons’ knowledge regarding OTC drugs in patients with CKD.

Examination questions	Pre-intervention score Median (IQR)	Post-intervention score Median (IQR)	*p* value
Risk factors (max: 3)	2 (1)	3 (0)	< 0.001 ^a^
Pharmacotherapy (max: 4)	3 (0)	4 (0)	< 0.001 ^a^
Unsuitable OTC drugs (max: 4)	2 (1)	4 (0)	< 0.001 ^a^

CKD, chronic kidney disease; IQR, interquartile range; OTC, over-the-counter; max, maximum.

^a^ Wilcoxon signed-rank test.

## 4 Discussion

In this study, we showed that registered salespersons had low levels of awareness and knowledge regarding OTC drug use in patients with CKD. The pharmacist intervention increased the participants’ awareness of the need to check renal function when selling OTC drugs that should be used cautiously by this population.

Training programs for healthcare professionals both before and after registration are very important to maintain public health and the safety of self-medication. Nevertheless, there is no specified educational program to prepare for the examination to become a registered salesperson in Japan. Thus, almost all candidates to become registered salespersons have to gain knowledge by self-learning. Additionally, the registered salesperson’s license is valid for life after passing the examination. In this study, we demonstrated that the participating registered salespersons had little knowledge about the appropriate use of OTC drugs in patients with renal disease (Tables 4 and 5). This finding suggests that a specific educational program for registered salespersons before registration might be needed to guarantee quality service to patients.

NSAIDs have been associated with progression of CKD [11] and onset of acute kidney injury [12]. Abnormal renal function is known to be a risk factor for hypermagnesemia and hyperaluminemia secondary to administration of magnesium and aluminum, both of which are major active ingredients of OTC antacids [13,14]. Thus, these OTC drugs should be avoided in patients with CKD [8]. However, patients with CKD also use NSAIDs frequently without requesting advice from community pharmacy staff [15]. Appropriate advice from pharmacy staff and other healthcare professionals is needed to ensure the safe use of OTC drugs [16,17]. Pai et al. mentioned that community pharmacists play a pivotal role in NSAID avoidance [7]. However, the results of our study indicate that a large proportion of registered salespersons had not recognized the need to check renal function status when selling NSAIDs and antacids despite having sold these drugs in Japan. These findings suggest the possibility that registered salespersons sometimes sell unsuitable OTC drugs to patients with CKD without checking their renal function status.

In our study, the registered salespersons’ awareness regarding OTC drug use in patients with CKD was increased after the intervention. It has been reported that the awareness of healthcare professionals influences their behavior [18–21]. These findings suggest that the pharmacist intervention might change the registered salespersons’ behavior: after the intervention, most registered salespersons (97.8%) recognized that checking renal function status was necessary when selling NSAIDs and antacids to patients with CKD. Furthermore, we performed a subgroup analysis to evaluate the relationship between the registered salespersons’ level of working experience and their awareness and/or behavior (S2 Table). In general, healthcare professionals’ experience level affects their behavior: more experienced healthcare professionals tend to perform checks more cautiously. Indeed, before the intervention, proportionally fewer registered salespersons with little experience (<5 years working experience, n = 8, 15.9%) than more experienced registered salespersons (n = 38, 30.2%) recognized the need to check the renal function status when selling NSAIDs, and this difference was significant (p < 0.05). However, a significant difference between the two groups was not observed in the post-intervention survey. These results suggest that the pharmacist intervention might change the behavior of registered salespersons of all experience levels.

We observed that the registered salespersons’ knowledge scores after the intervention were higher than those before the intervention, both in overall and for each section. These results suggest that the pharmacist intervention improved the registered salespersons’ knowledge about OTC drug use in patients with CKD. In Japan, all unsuitable OTC drugs for patients with CKD are contraindicated for patients with renal insufficiency. Therefore, registered salespersons should understand the epidemiology and risk factors of CKD to check whether patients have renal insufficiency. However, awareness of CKD is low among patients with kidney disease in primary care, particularly compared with their awareness of other chronic diseases, such as diabetes and hypertension [22]. Therefore, we examined the participants’ knowledge about the risk factors for CKD. After the intervention, most registered salespersons (97.8%) completely understood the risk factors for CKD. Furthermore, registered salespersons need to have topical knowledge to provide appropriate OTC drugs to customers with CKD. As shown by the participants’ improvement in knowledge scores after the intervention, the findings of this study suggest that the pharmacist intervention educational program is necessary to increase registered salespersons’ level of attention to OTC drug use in patients with CKD. Indeed, after the intervention, registered salespersons were able to determine whether OTC drugs were unsuitable for patients with CKD.

This study has some limitations. First, this study’s data are subject to sampling bias associated with the methods used. The survey and intervention in this study were conducted via continuation training for registered salespersons by the Kagoshima Pharmaceutical Association. Almost all members of the Kagoshima Pharmaceutical Association work in community pharmacies that dispense prescription medicine. Thus, the continuation training was advertised only to registered salespersons who worked with members of this association. As a result, most participating registered salespersons were women who worked in such pharmacies. Therefore, the participating registered salespersons worked under the supervision of community pharmacists. Although most registered salespersons worked with pharmacists, many had low awareness and knowledge about OTC drug use in patients with CKD. Therefore, this study might have overestimated the registered salespersons’ awareness, knowledge, and precaution implementation. Additionally, OTC drugs for urgent use, such as NSAIDs and antacids, might be commonly purchased in shops where nonpharmacist salespersons work alone, such as drug stores. Thus, it is important to clarify the effects of the pharmacist intervention on registered salespersons who work in drug stores. In our study, the participants’ knowledge scores after the intervention were higher than those before the intervention in the subgroup analysis of registered salespersons who work in drug stores (shown in S3 Table). Although the sample size was small, these results suggest that the pharmacist intervention might improve knowledge about OTC drug use by patients with CKD among registered salespersons who work without pharmacists. Second, this study did not measure the participants’ educational background. When this research was performed in January 2015, a high school diploma was necessary to take the qualifying examination to become a registered salesperson. Therefore, all participants had at least graduated from high school. However, there is no more information about the participants’ educational background. Differences in educational background might affect registered salespersons’ behavior. Furthermore, the requirement of a high school diploma to take the examination was removed in April 2015. Therefore, it is necessary to clarify the influence of educational background. Third, this study did not evaluate whether the registered salespersons’ awareness and knowledge were maintained after the study was complete. In general, continuous training is needed to maintain the knowledge of healthcare professionals, including pharmacy staff [23, 24]. Additionally, a Japanese ministerial ordinance obligates registered salespersons’ employers to provide continuous education programs for their employees. We therefore believe that such pharmacist interventions and surveys should be performed periodically. Although further study will be needed, these results emphasize the importance of continuing education programs for registered salespersons regarding OTC drug use in patients with CKD.

## 5 Conclusions

We investigated Japanese nonpharmacist salespersons’ awareness, knowledge, and implementation of precautions regarding OTC drug use in patients with CKD. Additionally, we evaluated the effects of a pharmacist intervention that was intended to improve registered salespersons’ knowledge and awareness of this topic. We found that before the intervention, registered salespersons had low awareness and knowledge about OTC drug use by patients with CKD. However, we also found that the pharmacist intervention improved both awareness and knowledge. We believe that continuing education programs for registered salespersons are necessary to prevent inappropriate OTC drug use by patients with CKD.

## Supporting information

S1 File The questionnaire in this study (English version). (DOCX)

S1 Table Lecture contents. (DOCX)

S2 Table Comparisons of awareness and behavior regarding OTC drug use in patients with CKD between registered salespersons with little working experience (<5 years) and more experienced registered salespersons. (DOCX)

S3 Table Knowledge regarding use of OTC drugs by patients with CKD among registered salespersons who work without pharmacists. (DOCX)
