# Supplementary material for: Knowledge and awareness of nonpharmacist salespersons regarding over-the-counter drug use in patients with chronic kidney disease in Japan
Source: PLoS One. 2019 Mar 20;14(3):e0213763. doi: 10.1371/journal.pone.0213763 (PMC6426248; doi:10.1371/journal.pone.0213763)
Supplement: S1 File — (DOCX) [file pone.0213763.s001.docx]

**Supporting file 1: The questionnaire in this study (English version)**

The results of this survey will only be used for an academic article and an academic conference presentation. The personal information is not used for any other purpose. The data will be analyzed anonymously. In addition, all information will be destroyed appropriately when this study has been completed. Please answer the questionnaire if you agree with the above.

If you have any questions, please contact us.

1. Seat Number No.
2. Gender □　Male □　Female
3. Age 　　　　　years
4. Work experience (If you have less than 1 year of work experience, answer “0.”)

　　　　　years

1. What type of business is your workplace?

□　Pharmacy (deals with prescription medicine)

□　Drug store (does not deal with prescription medicine)

□　Household distribution □　Other ( )

1. Which types of goods are dispensed in your workplace? (Multiple answers allowed)

□　OTC drugs (Class 1, Class 2, Class 3) □　OTC drugs (Drugs requiring guidance)

□　Prescription drugs □　Quasi-drugs (e.g., cough drops) □　Cosmetics

□　Functional foods and dietary supplements　□　Medical devices

□　Other (　　　　　　　　　　　　　)

1. Do you have experience with selling antacids?

□　Yes □　No □　Unknown

↓

If you chose “Yes”

Please give the names of antacids you have sold, if you remember. (Multiple answers allowed)

( )

1. Do you have experience with selling NSAIDs?

□　Yes □　No □　Unknown

↓

If you chose “Yes”

Please give the names of NSAIDs you have sold, if you remember. (Multiple answers allowed)

( )

1. Which of the following are important points to check when you sell antacids? **Please choose 3 answers.**

- Hypertension □　Bronchial asthma
- Long-term use
- Kidney disease □　Liver disease □　Drinking □　Smoking
- Whether the customer is using other concomitant medications

1. Which of the following are important points to check when you sell NSAIDs? **Please choose 3 answers.**

- Gastrointestinal disease □　Aspirin induced bronchial asthma
- Long-term use
- Kidney disease □　Liver disease □　Drinking □　Smoking
- Whether the customer is using other concomitant medications

1. Please indicate how important the following services are to avoid adverse drug events caused by OTC drugs.
2.
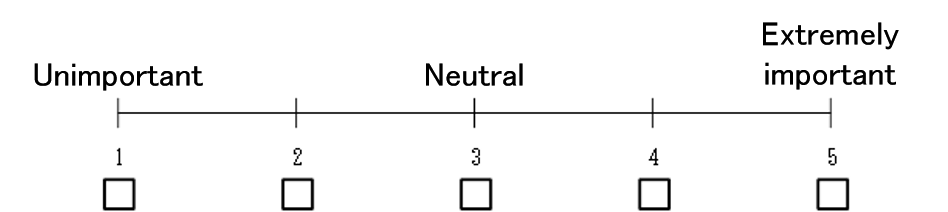
Checking renal function when selling OTC drugs
3.
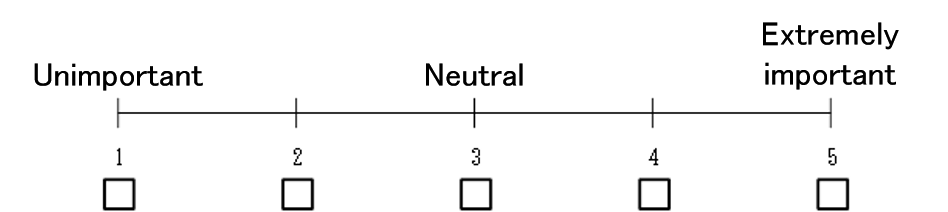
Considering renal function when providing pharmaceutical information
4.
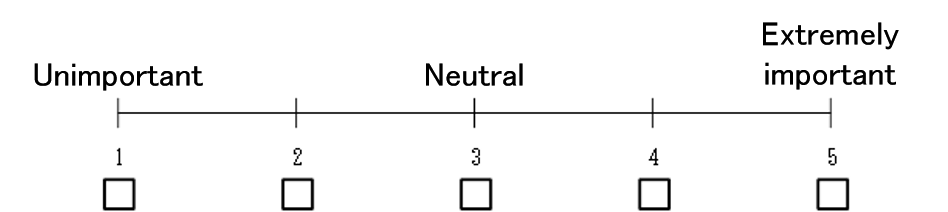
 Monitoring of drug-induced renal dysfunction during long-term OTC drug use
5. Patients with renal dysfunction are less likely to have adverse drug events than patients with normal renal function. Instead, drugs can lack efficacy in patients with renal dysfunction.

□　True □　False

1. Renal function can be estimated by a blood test.

□　True □　False

1. Patients with renal dysfunction should also be aware of health foods and supplements.

□　True □　False

1. What is the prevalence of chronic kidney disease in the Japanese adult population?
   - One out of seven hundred □　One out of seventy □　One out of seven
2. Renal dysfunction should be suspected in patients with which of the following backgrounds? **Please choose 3 answers.**

- Depression □　Liver disease □　Diabetes
- Hypertension □　Crohn’s disease □　Elderly (Age over 75 years)

1. Which of the following drugs/functional foods and dietary supplements require checks for renal dysfunction when renal dysfunction should be suspected in the patient?

- Acinon^®^Z (Nizatidine) □　Ohta-isan (containing aluminum silicate)
- Surazin^®^ (Chlorpheniramine maleate) □　Bayer aspirin^®^ (aspirin)
- Nephvitan KD (containing folic acid) □　Ao-jiru (containing kale)
- Tylenol^®^ A (acetaminophen) □ Drewell^®^ (Diphenhydramine hydrochloride)
