# Supplementary material for: Knowledge and awareness of nonpharmacist salespersons regarding over-the-counter drug use in patients with chronic kidney disease in Japan
Source: PLoS One. 2019 Mar 20;14(3):e0213763. doi: 10.1371/journal.pone.0213763 (PMC6426248; doi:10.1371/journal.pone.0213763)
Supplement: S1 Table — (DOCX) [file pone.0213763.s002.docx]

**Supporting table 1 Lecture contents**

| The role of the kidney | - - - Filtering waste materials, such as toxic substances     - Regulating blood pressure and mineral balance     - Producing hormones to form red blood cells and bone     - Maintaining fluid balance |
| --- | --- |
| About Chronic Kidney disease | - - - The definition of CKD     - The prevalence of CKD in Japan   (one out of seven in the adult population)   - - - Risk factors for renal dysfunction   (Old age, diabetes, hypertension)   - - - The meaning of serum creatinine     - Factors to suspect CKD in patients at pharmacies and drug stores   (Using information about concomitant drugs, such as phosphate binders and spherical carbon adsorbent drugs) |
| Drug use in patients with renal insufficiency | - - - Pharmacokinetic changes in patients with renal insufficiency     - The risk of adverse drug events in in patients with renal insufficiency     - (Renal insufficiency is a risk factor for adverse drug events)     - OTC drugs that are unsuitable for patients with CKD   (H_2_ blockers, aluminum, magnesium, functional foods and dietary supplements containing high levels of potassium, NSAIDs) |
