# Supplementary material for: Knowledge and awareness of nonpharmacist salespersons regarding over-the-counter drug use in patients with chronic kidney disease in Japan
Source: PLoS One. 2019 Mar 20;14(3):e0213763. doi: 10.1371/journal.pone.0213763 (PMC6426248; doi:10.1371/journal.pone.0213763)
Supplement: S2 Table — (DOCX) [file pone.0213763.s003.docx]

**Supporting table 2. Comparisons of awareness and behavior regarding OTC drug use in patients with CKD between registered salespersons with little working experience (<5 years) and more experienced registered salespersons**

**Pre-intervention**

| **Medicine** | **Little experienced**  **n (%)** | **More experienced**  **n (%)** | ***p* value** |
| --- | --- | --- | --- |
| NSAIDs  Chose to check renal function | 8 (15.9) | 38 (30.2) | < 0.05 ^a^ |
| Antacids  Chose to check renal function | 22 (41.5) | 63 (50) | 0.33 ^a^ |

**Post-intervention**

| **Medicine** | **Little experienced n (%)** | **More experienced n (%)** | ***p* value** |
| --- | --- | --- | --- |
| NSAIDs  Chose to check renal function | 52 (98.1) | 123 (97.6) | 1.00 ^a^ |
| Antacids  Chose to check renal function | 53 (100) | 122 (96.8) | 0.32 ^a^ |

CKD, chronic kidney disease; NSAID, nonsteroidal anti-inflammatory drug.

^a^ Fisher’s exact test.
