# Supplementary material for: Knowledge and awareness of nonpharmacist salespersons regarding over-the-counter drug use in patients with chronic kidney disease in Japan
Source: PLoS One. 2019 Mar 20;14(3):e0213763. doi: 10.1371/journal.pone.0213763 (PMC6426248; doi:10.1371/journal.pone.0213763)
Supplement: S3 Table — (DOCX) [file pone.0213763.s004.docx]

**Supporting table 3. Knowledge regarding use of OTC drugs by patients with CKD among registered salespersons who work without pharmacists**

| **Examination questions** | **Pre-intervention score**  Median (IQR) | **Postintervention score**  Median (IQR) | ***p* value** |
| --- | --- | --- | --- |
| Risk factors (max: 3) | 2 (1) | 3 (0) | 0.13 ^a^ |
| Pharmacotherapy (max: 4) | 3 (1) | 4 (0) | < 0.05 ^a^ |
| Unsuitable OTC drugs (max: 4) | 2 (1) | 4 (1) | < 0.05 ^a^ |
| Total score (max: 11) | 8 (3) | 11 (1) | < 0.05 ^a^ |

CKD, chronic kidney disease; IQR, interquartile range; OTC, over-the-counter; max, maximum.

^a^ Wilcoxon signed-rank test.
